# 7,8-Dihydroxiflavone Protects Adult Rat Axotomized Retinal Ganglion Cells through MAPK/ERK and PI3K/AKT Activation

**DOI:** 10.3390/ijms221910896

**Published:** 2021-10-08

**Authors:** Caridad Galindo-Romero, Beatriz Vidal-Villegas, Javier Asís-Martínez, Fernando Lucas-Ruiz, Alejandro Gallego-Ortega, Manuel Vidal-Sanz

**Affiliations:** 1 Departamento de Oftalmología, Campus de CC de la Salud, Universidad de Murcia e Instituto Murciano de Investigación Biosanitaria (IMIB) Virgen de la Arrixaca, El Palmar, 30120 Murcia, Spain; beatrizvidalvillegas@gmail.com (B.V.-V.); javier.asism@um.es (J.A.-M.); fernando.lucas@um.es (F.L.-R.); alejandrogallego@um.es (A.G.-O.); manuel.vidal@um.es (M.V.-S.); 2 Servicio de Oftalmología, Hospital Clínico San Carlos, Instituto de Investigación Sanitaria del Hospital Clínico San Carlos (IdISSC), 28040 Madrid, Spain

**Keywords:** axotomy, adult rats, intraorbital optic nerve transection, retinal ganglion cells, 7,8-dihydroxiflavone (DHF), 7,8-dihydroxiflavone/TrkB signaling, AKT, MAPK, tropomyosin related kinase B (TrkB)

## Abstract

We analyze the 7,8-dihydroxyflavone (DHF)/TrkB signaling activation of two main intracellular pathways, mitogen-activated protein kinase (MAPK)/ERK and phosphatidylinositol 3 kinase (PI3K)/AKT, in the neuroprotection of axotomized retinal ganglion cells (RGCs). Methods: Adult albino Sprague-Dawley rats received left intraorbital optic nerve transection (IONT) and were divided in two groups. One group received daily intraperitoneal DHF (5 mg/kg) and another vehicle (1%DMSO in 0.9%NaCl) from one day before IONT until processing. Additional intact rats were employed as control (n = 4). At 1, 3 or 7 days (d) after IONT, phosphorylated (p)AKT, p-MAPK, and non-phosphorylated AKT and MAPK expression levels were analyzed in the retina by Western blotting (n = 4/group). Radial sections were also immunodetected for the above-mentioned proteins, and for Brn3a and vimentin to identify RGCs and Müller cells (MCs), respectively (n = 3/group). Results: IONT induced increased levels of p-MAPK and MAPK at 3d in DHF- or vehicle-treated retinas and at 7d in DHF-treated retinas. IONT induced a fast decrease in AKT in retinas treated with DHF or vehicle, with higher levels of phosphorylation in DHF-treated retinas at 7d. In intact retinas and vehicle-treated groups, no p-MAPK or MAPK expression in RGCs was observed. In DHF- treated retinas p-MAPK and MAPK were expressed in the ganglion cell layer and in the RGC nuclei 3 and 7d after IONT. AKT was observed in intact and axotomized RGCs, but the signal intensity of p-AKT was stronger in DHF-treated retinas. Finally, MCs expressed higher quantities of both MAPK and AKT at 3d in both DHF- and vehicle-treated retinas, and at 7d the phosphorylation of p-MAPK was higher in DHF-treated groups. Conclusions: Phosphorylation and increased levels of AKT and MAPK through MCs and RGCs in retinas after DHF-treatment may be responsible for the increased and long-lasting RGC protection afforded by DHF after IONT.

## 1. Introduction

Retinal ganglion cells (RGCs) are the only neurons of the retina that send the visual information through the optic nerve to the retinorecipient areas of the brain. Among all experimental models to study neuronal degeneration and neuroprotection, intraorbital optic nerve transection (IONT) is widely used due to its high reproducibility and easy access to RGC axons compared to other central nervous system (CNS) tracts. This model has been used frequently both in rats [1,2,3,4,5] and mice [5,6,7,8,9,10], showing two phases of RGC degeneration: a first fast phase, from 1 to 9 days after IONT in mice [7,11] or 14 in rats [2,4,5], leaving 15% of the RGC population alive; and a second slower phase, from 9 to 45 days or longer, in which RGCs degenerate much slower [5].

Neuroprotective therapies aim to restore visual function by preventing RGC degeneration using a variety of neuroprotective compounds, including trophic and neurotrophic factors [5,12,13,14,15], alpha-2 adrenergic receptors agonist [16,17] nitric oxide (NOS) inhibitors, or controlling glia-mediated neuroinflammation (reviewed in [18]).

The neurotrophin brain-derived neurotrophic factor (BDNF) is one of the most efficient RGC neuroprotectants [7,11,14,19,20,21,22,23] and has been used in several experimental models such as IONT [7,9], intraorbital optic nerve crush (IONC) [24], ocular hypertension (OHT) [14,25], or in an in vivo model of focal light emitting diode-induced cone-photoreceptor phototoxicity [23,26]. When administered intravitreally at the time of IONT, BDNF rescues degenerating RGCs during the first phase of RGC death [5,7,11,21,22]; however, this effect is transient. BDNF binds and activates its high-affinity tropomyosin-related kinase B (TrkB) receptor; triggers its dimerization, autophosphorylation, and internalization; and subsequently activates downstream signaling intracellular cascades: phosphatidylinositol-3 kinase (PI3-K) through its main effector of neuronal survival, serine/threonine kinase (AKT, or protein kinase B); mitogen-activated protein kinase (MAPK)/ERK and phospholipase-C (PLC-Υ) [27,28,29]. Each subpathway triggers a cascade of phosphorylation and dephosphorylation of several molecules that culminate in the activation of transcription factors that promote gene expression of elements that support cell survival. For instance, the PI3K/AKT pathway promotes anti-apoptotic signaling and pro-survival activity and regulates N-methyl-D-aspartic acid (NMDA) receptor-dependent synaptic plasticity [30,31,32]. MAPK/ERK pathways activated by BDNF seem to be relevant for local protein synthesis involved in prolonged increase in synaptic transmission, essential in neuronal plasticity and long-term potentiation [33], and they have an important role in neuronal growth and differentiation [34].

The flavonoid 7,8-dihydroxyflavone (DHF) is a small molecule agonist of TrkB [35] and, thus, a mimetic of the neurotrophin BDNF [36,37] that has been studied recently as a neuroprotective drug in a number of experimental models of CNS diseases [36,38,39] (for review see [40]). In the retina, the effect of DHF has been studied in few experimental models: against excitotoxicity in isolated RGCs [41], or hypoxic-ischemic injury [42], both in vitro. In vivo, in a model of chronic intermittent hypoxia in the retina, DHF reduces the production of reactive oxygen species (ROS), activates TrkB signals and downstream AKT and ERK signaling pathways, and upregulates the expression of mature BDNF, alleviating RGC damage [43]. DHF has been also shown to have antioxidant and anti-inflammatory effects (reviewed in [44]).

We have recently documented that DHF has a potent in vivo neuroprotective effect for RGCs against IONT, at an optimal dose of 5 mg/kg administered intraperitoneally [45]. The percentages of surviving Brn3a^+^RGCs in vehicle- or DHF-treated rats one week after IONT were 60 and 94%, respectively [45]. The DHF afforded neuroprotection was observed up to three weeks after IOP and, thus, lasted longer than our previously reported studies in which we administered a single intravitreal injection of BDNF after IONT [22] or crush [7,24]. DHF is known to activate TrkB receptor through its phosphorylation [40,43,46], and in our study the retinas treated with DHF showed a higher survival of RGCs and TrkB phosphorylation (pTrkB) at 7 days, thus suggesting that DHF reaches the retina and activates TrkB [45]. However, the signaling pathways responsible for the in vivo neuroprotective effects of DHF in the axotomized adult rat retina are, to the best of our knowledge, unknown. Moreover, whether different neuronal and nonneuronal retinal cells are involved in the neuroprotection process is also unknown.

For the present studies, using an in vivo rat retinal model of axonal damage, we demonstrate for the first time that the systemic administration of DHF results in activation of the main TrkB activated signaling pathways: (i) phosphatidylinositol 3-kinase/protein kinase B (PI3K/AKT), and (ii) mitogen activated protein kinases extracellular signal regulated kinases 1 and 2 (MAPK/ERK; ERK1/2), thus suggesting their involvement in the DHF afforded neuroprotection for RGCs against IONT. Moreover, our immunohistochemical studies suggest an important role for RGCs and Müller cells as effectors in the neuroprotection process.

## 2. Results

### 2.1. DHF Treatment Activates MAPK Signaling

#### 2.1.1. MAPK Protein Levels and Expression Pattern

**Figure 1:**
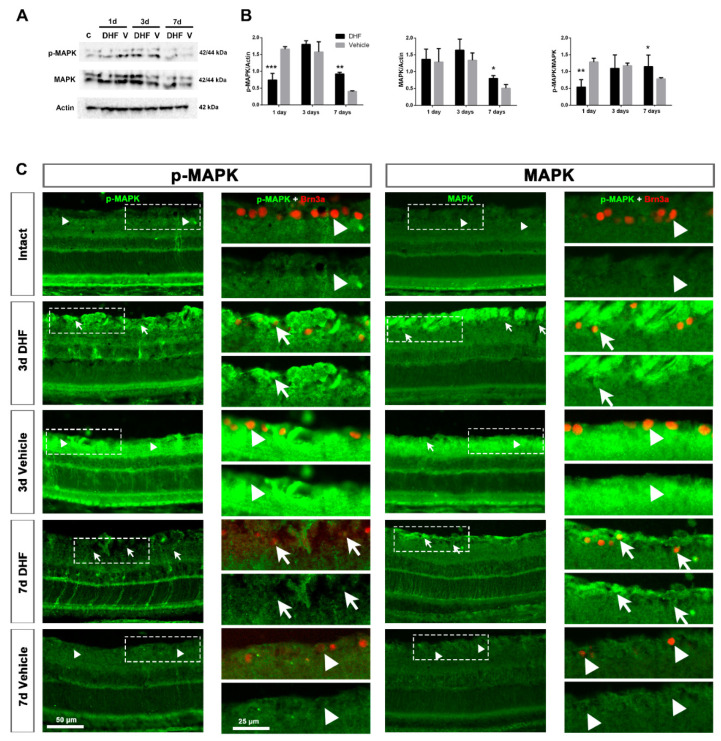
MAPK signaling pathway. Representative images of Western blot (**A**) from MAPK protein levels in retinal samples (pool of four samples/time-point). Western blot was performed with retinal extracts from intact (c, control) and left injured retinas treated daily with DHF or vehicle (V) analyzed at 1, 3 or 7d after IONT. (**B**): Bar graphs of the quantitative analysis of phosphorylated (*p*-) MAPK and the total MAPK protein compared to actin protein levels, and normalized p-MAPK/MAPK. In all graphs, results are presented as mean ± SD relative to intact retinas (considered 1.0). Kruskal–Wallis test, * *p* < 0.05, ** *p* < 0.01, *** *p* < 0.001). (**C**): Representative cross-sectioned retinas (20×) of p-MAPK (left) and total MAPK (right) signal in intact and axotomized retinas treated with DHF or vehicle at 3 and 7 days after IONT. In the right column of each one, a magnification of double immunostaining with Brn3a (upper part) and p-MAPK or MAPK alone (lower part) is shown, pointing the co-expression (arrows) and no co-expression (arrowheads) between them. RNFL = retinal nerve fiber layer, GCL = ganglion cell layer, IPL = inner plexiform layer, INL = inner nuclear layer, OPL = outer plexiform layer, ONL = outer nuclear layer, PRL = photoreceptor layer, RPE = retinal pigment epithelium, d = days post-lesion. Scale bar is shown at the bottom left.

Western blotting analysis showed an increase in the amount of total MAPK protein from 1 day after IONT (Figure 1A,B), with a strong phosphorylation from 1 to 3 days after IONT in vehicle-treated groups and from 3 days in DHF-treated groups. At 7 days, MAPK protein level decreased, but the activation was significantly higher in DHF-treated groups than in vehicle-treated ones.

To analyze the MAPK signal and location in the retina, micrographs were acquired with the same settings. In intact retinas, there was a weak signal of both p-MAPK and total MAPK proteins along the retina, mainly observed in the outer retina, in the photoreceptor layer (PRL) and retinal pigment epithelium (RPE) (Figure 1C). The levels of p-MAPK and MAPK increased significantly 3 days after IONT, both in DHF- or vehicle-treated retinas, as was also observed in Western blots (Figure 1A,C). This activation was observed in the ganglion cell layer (GCL), in the outer retina and across the retina. At 7 days, this signal was significantly higher in DHF-treated retinas when compared to vehicle-treated groups.

#### 2.1.2. MAPK Activation in Retinal Ganglion Cells

Since DHF-treated retinas showed higher RGC survival [45], we investigated whether MAPK was activated in RGCs. In intact retinas, no signal of p-MAPK or MAPK was observed in Brn3a-labeled RGCs (Figure 1C, arrowheads). The time-course of RGC degeneration was slower in DHF-treated retinas at 3 and 7 days after IONT when compared to vehicle-treated ones. P-MAPK and MAPK were located in the retinal nerve fiber layer (RNFL) and in the RGC nuclei of DHF-treated animals (Figure 1C, white arrows), but no p-MAPK and MAPK signals were observed in RGCs of vehicle-treated ones (Figure 1C, arrowheads).

#### 2.1.3. MAPK Activation in Müller Cells

**Figure 2:**
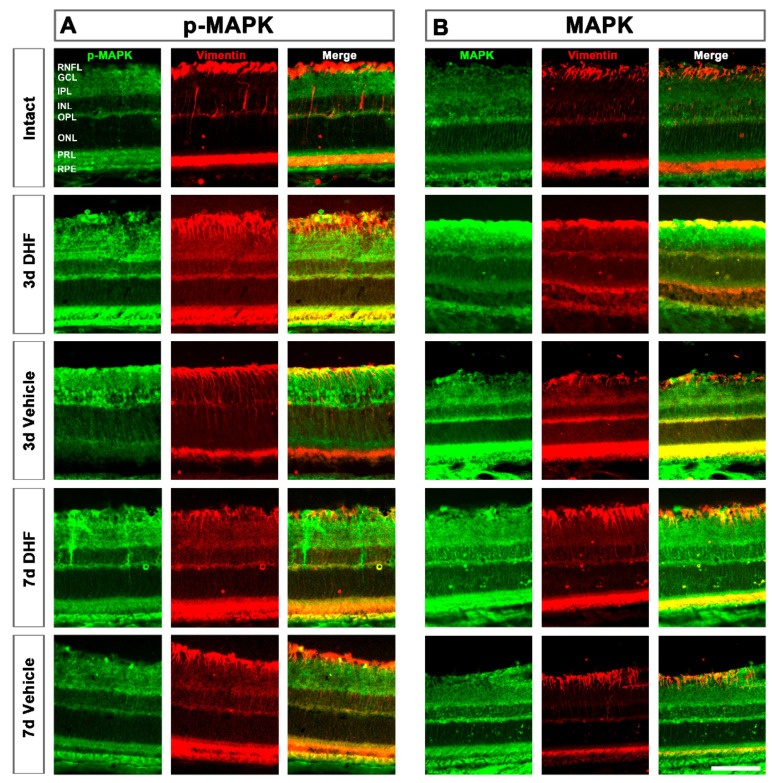
Activation of MAPK signaling pathway in Müller cells (MCs). Representative micrographs (20×) of phosphorylated (p-) MAPK (**A**) and total MAPK (**B**) signal in intact, and axotomized retinas treated with DHF or vehicle at 3 and 7 days after IONT. The signal of each protein, MCs immunodetected with vimentin and the co-localization of both images are shown. RNFL = retinal nerve fiber layer, GCL = ganglion cell layer, IPL = inner plexiform layer, INL = inner nuclear layer, OPL = outer plexiform layer, ONL = outer nuclear layer, PRL = photoreceptor layer, RPE = retinal pigment epithelium. Scale bar at the bottom right = 50 µm.

After IONT, vimentin immunodetection revealed a strong hypertrophy of Müller cells (MCs) expressing higher amounts of p-MAPK (Figure 2, left) and MAPK (Figure 2, right). At 3 days after IONT, in DHF-treated retinas we observed colocalization of p-MAPK with MCs throughout the retina. Colocalization of the total form of MAPK and MCs was only present in the GCL. In the vehicle-treated retinas we observed the opposite; p-MAPK was located in the GCL whereas MAPK was present throughout the whole retina. While at 3 days the increased MAPK was observed in both DHF- and vehicle-treated groups, at 7 days the phosphorylation of p-MAPK was higher in DHF-treated groups.

### 2.2. DHF Treatment Activates PI3K/AKT Signaling Pathway

#### 2.2.1. AKT Protein Levels and Expression Pattern

**Figure 3:**
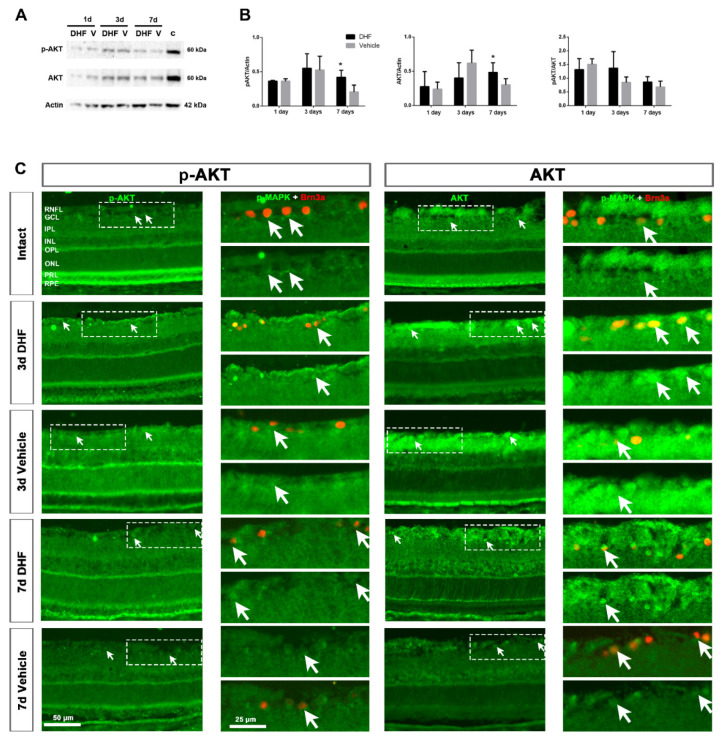
AKT signaling pathway. Representative images of Western blot (**A**) from AKT protein level in retinal samples (pool of four samples/time-point). Western blot was performed with retinal extracts from intact (c) and left injured retinas daily treated with DHF or vehicle (V) analyzed at 1, 3 or 7d after IONT. (**B**): Bar graphs of the quantitative analysis of phosphorylated (*p*-) AKT and the total protein AKT compared to actin protein level, and normalized p-AKT/AKT. In all graphs, results are presented as mean ± SD relative to intact retinas (considered 1.0). Kruskal–Wallis test, * *p* < 0.05). (**C**): Representative cross-sectioned retinas (20×) of phosphorylated AKT (left) and total AKT (right) signal in intact and axotomized retinas treated with DHF or vehicle at 3 and 7 days after IONT. In the right column of each one, a magnification of double immunostaining with Brn3a (upper part) and p-AKT or AKT alone (lower part) is shown, pointing the co-expression (arrows) and no co-expression (arrowheads) between them. RNFL = retinal nerve fiber layer, GCL = ganglion cell layer, IPL = inner plexiform layer, INL = inner nuclear layer, OPL = outer plexiform layer, ONL = outer nuclear layer, PRL = photoreceptor layer, RPE = retinal pigment epithelium, d = days post-lesion. Scale bar is shown at the bottom left.

Western blotting analysis showed that AKT protein level was strong in intact retinas (Figure 3A). After IONT, both p-AKT and AKT decreased at 1 day and then increased at 3 days in both DHF- and vehicle-treated groups. At 7 days, the amount of p-AKT and AKT decreased, and it was significantly higher in DHF-treated groups compared to vehicle-treated ones, although normalized p-AKT/AKT did not show significant differences compared to vehicle-treated groups (Figure 3A,B). In intact retinas, immunohistochemistry showed that this strong signal of p-AKT and AKT was found in the outer retina, in the outer segments of the PRs and RPE (Figure 3C). In both groups, 3 days after IONT, the levels of total and p-AKT were lower in the PRL and RPE but higher across the retina, mainly in the retinal fiber layer (RNFL), GCL and inner nuclear layer (INL). At 7 days, the signal pattern was similar to what we observed in the Western blotting; there was more AKT and p-AKT signal in DHF-treated retinas compared to vehicle-treated ones (Figure 3C).

#### 2.2.2. AKT Activation in Retinal Ganglion Cells

In intact retinas, few RGCs express low levels of p-AKT and AKT (Figure 3C). At 3 and 7 days after IONT, p-AKT and AKT signals co-localized with Brn3a^+^RGCs in DHF- and vehicle groups. This signal was slightly higher in the RGC nuclei when retinas were treated with DHF (arrows) at 3 and 7 days after IONT (Figure 3C).

#### 2.2.3. AKT Activation in Müller Cells

**Figure 4:**
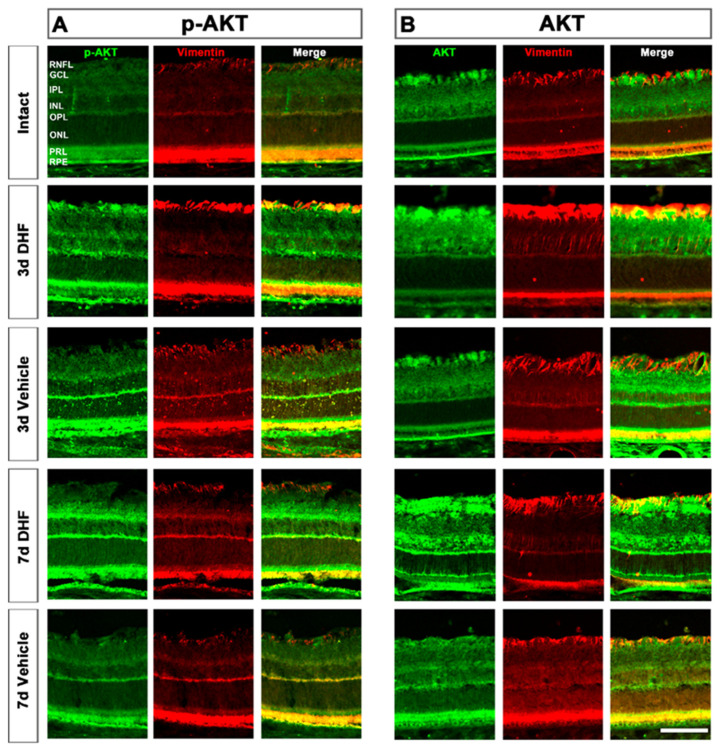
Activation of AKT signaling pathway in Müller cells (MCs). Representative micrographs (20×) of phosphorylated AKT (**A**) and total AKT (**B**) protein levels in intact, and axotomized retinas treated with DHF or vehicle at 3 and 7 days after IONT. The signal of each certain protein, MCs immunodetected with vimentin and the co-localization of both images are shown. RNFL = retinal nerve fiber layer, GCL = ganglion cell layer, IPL = inner plexiform layer, INL = inner nuclear layer, OPL = outer plexiform layer, ONL = outer nuclear layer, PRL = photoreceptor layer, RPE = retinal pigment epithelium. Scale bar at the bottom right = 50 µm.

Colocalization between AKT and vimentin^+^MCs showed a similar behavior as observed with MAPK signaling pathway (Figure 2). MCs showed high levels of phosphorylation from 3 days after IONT and treatment with DHF or vehicle compared to intact retinas. At 7 days, the increased AKT protein level and activation was maintained higher when DHF treatment was administered (Figure 4).

## 3. Discussion

The results of this study indicate that systemic treatment with DHF prevents axotomized RGC degeneration through the activation of the DHF/TrkB signaling pathway, specifically PI3K/AKT and MAPK/ERK signaling pathways. This is the first in vivo study demonstrating that the phosphorylation of MAPK/ERK and AKT induced by DHF treatment is located in RGCs and MCs, thus establishing a neuro-glial interaction that may be responsible for the DHF-afforded long-term RGC protection against axonal damage.

DHF is an agonist of the TrkB receptor. Both BDNF/TrkB and DHF/TrkB signaling pathways have been demonstrated to have a role in inhibiting apoptosis and, thus, protecting RGCs against retinal damage [42,43,47,48]. A recent study from our group showed strong rescuing effects of DHF against axotomy of the RGC population with a systemic daily dose of 5 mg/kg; almost the entire population of axotomized RGCs survived by 7 days (see Figures 3 and 6 of [45]), and the protection lasted for three weeks, considerably longer than our previously observed effects when a single intravitreal injection of 5 µg BDNF was administered as neuroprotectant [7,22]. It is likely that such in vivo differences in the protective lasting effects result from different pharmacokinetics of the receptor TrkB activation by the ligands BDNF or 7,8-DHF, which may follow different temporal patterns [36,47]. DHF is a small molecule (254 Da) [44], approximately 1% compared to the size of BDNF (28 kDa), that crosses the blood–brain barrier [46]. This difference in size allows DHF to have a long half-life in plasma and to reach the brain within 10 min of administration [38], and it has no apparent toxicity when administered chronically [45,49]. Indeed, BDNF-elicited TrkB signals are transient and fade within 1h [29] while TrkB activation by DHF may last for hours [36,43,45]. Moreover, BDNF-activated TrkB receptors, but not DHF-activated TrkB receptors, are ubiquinated and degraded [36].

TrkB receptor, activated by neurotrophins, is followed by the downstream activation of PI3K/AKT and MAPK/ERK pathways, which are the two strong survival pathways that block both extrinsic and intrinsic apoptotic pathways [20,50]. In agreement with our recent report [45], DHF treatment resulted in an increased Brn3a^+^RGC survival when compared to vehicle-treatment at 3 and 7 days after IONT. This finding was paralleled by increased levels of both total MAPK and phosphorylated MAPK proteins at a time when RGC survival was optimal. These results are similar to those observed in other experimental models, such as chronic intermittent hypoxia, where DHF treatment activates downstream AKT and ERK signaling pathways in RGCs in vitro [43] or, with BDNF treatment, only from 1h to 3 days after IONT [29].

MAPK/ERK is involved in cellular growth and differentiation by activating three members—P38, ERK1/2 and JNK—and it has been described to have a long-term effect in injured retinas. In this study, we found that the amount of MAPK was higher from 1 day after IONT with both treatments, but phosphorylation was higher in the vehicle-treated group, which might mean that RGCs are not degenerating yet when DHF is administrated. After 3 days, DHF or vehicle treatment in normalized p-MAPK/MAPK levels showed a similar activation, and it remained highly phosphorylated at 7 days in retinas treated with DHF. This is in agreement with our previous description of a higher phosphorylation of TrkB at this time-point [45], which initiates this signaling cascade, as has been described with BDNF [47]. The late activation of p-MAPK and the maintenance of phosphorylation with DHF at 7 days could explain the longer survival rate of RGCs after IONT when compared to BDNF, which has a shorter effect [7,22]. It is interesting that p-MAPK levels increased but also the total MAPK protein, although further studies are needed to understand such an increased MAPK protein expression.

In this study, the results of DHF were compared with vehicle. Previous findings from our group have shown that the time course of RGC degeneration after IONT in rats without treatment [1,2,4] or with intraperitoneal injection of vehicle (1% DMSO in 0.9% NaCl) are comparable [45], suggesting that intraperitoneal administration of vehicle has no effect on its own in the IONT model. Thus, our results may be interpreted in that IONT induces this molecular change by itself.

Moreover, MAPK and p-MAPK proteins were clearly observed in DHF-treated surviving RGCs, while no MAPK nor p-MAPK signal was observed in intact or vehicle-treated retinas (Figure 1), similar to the results described with BDNF at shorter times [29]. MAPK and p-MAPK were also observed 3 days after IONT in MCs of retinas treated with DHF or vehicle, indicating that MCs respond fast to retinal damage by MAPK phosphorylation. This result, similar to a previous study that described MAPK activation after IONT without treatment [51], would suggest that MCs become active after injury to protect RGCs. However, the burst activation of MAPK in MCs was maintained up to 7 days in the DHF-treated group but decreased in the vehicle-treated group. Previous studies have described IONT induced MCs hypertrophy, with upregulation of GFAP and vimentin 7 days after optic nerve injury [25]. This overexpression is mainly observed in the GCL where the end feet of MCs reside [52] and close to the damaged RGCs, as shown in Figure 2 and Figure 4. Thus, it is tempting to suggest that the activation of the survival MAPK signaling pathway results in neuroprotection through activation of both MCs and RGCs, as has been described in other experimental models such as NMDA excitotoxicity [53,54], or retinal detachment, ischemia-reperfusion, inflammation and glaucoma (reviewed in [55]). P-MAPK and MAPK was also observed in the outer retina; however, further studies are need to understand this event.

We also studied the AKT signaling pathway. PI3-K activates AKT (protein kinase B, a serine/threonine kinase) and promotes cell survival and growth, inhibiting Bad and Bcl-2, which are anti-apoptotic. The phosphorylation of AKT also suppress Casp-9 and Forkhead (reviewed in [27]). Our results indicate that, in contrast to the MAPK/ERK signaling pathway, AKT is abundant in intact retinas, and it decreases after IONT. This was somewhat surprising because previous studies have reported higher AKT levels after an injury [42,43,51]. Histologically, immunohistochemistry revealed higher levels of both p-AKT and AKT in the outer segments of PRs and in the RPE of intact retinas. After IONT, the signal of p-AKT and AKT decreased in the outer retina but increased in the GCL and MCs. Although the signal intensity was weak, AKT was constitutively expressed in intact RGCs, and the results indicate that DHF-treated retinas had a stronger signal intensity in the RGC nuclei at 3 and 7 days after IONT. In the present experiments we have observed a large amount of AKT and p-AKT in the outer segments of photoreceptors in intact retinas, as well as p-MAPK and MAPK. Since these have large amounts of stress signals due to the continuous renewal of the PRs [56,57], it is likely that p-AKT and AKT are more increased in this section to protect the PRs [58,59]. When more severe damage occurs, such as IONT, it is conceivable that retinal tissue prioritizes damage and thus protects the most damaged neurons, in this case the RGCs, by phosphorylating AKT, although further studies are required to test this hypothesis.

Finally, the present study has some limitations. First, our results document that, following DHF administration, MAPK and AKT signaling pathways are activated, the two main signaling pathways of TrkB, but it remains to be further elucidated whether PLC-Υ and GTP-ases pathways are also implicated in DHF-afforded neuroprotection, and which of the members of each pathway are responsible for this neuroprotective effect. Moreover, further studies are needed to demonstrate that DHF-afforded neuroprotection could be blocked using specific MAPK and AKT inhibitors.

Secondly, DHF has been shown to protect against glutamate-induced toxicity in HT-22 cells via its antioxidant activity [60]. Moreover, DHF has been shown to have antioxidant effects and protects cells in vitro from apoptotic cell death induced by high glucose levels [61]. However, the protective effects of DHF against oxidative stress and excitotoxicity, such as those observed in classic excitotoxicity models, such as the intravitreal injection of NMDA (Gallego-Ortega et al., unpublished observations) and nonclassical excitotoxicity models [60], or after a transient ischemia of the retina induced by an acute elevation of the IOP (Gallego-Ortega et al., unpublished observations), needs to be further studied to confirm whether they follow the same signaling pathways. Thirdly, the macro- and micro-glial response caused by IONT and their importance in RGC survival has been well documented [7,25,62]. Here we show that MCs respond to axonal damage and treatment, and DHF has been shown to have an anti-inflammatory effect through microglial activation in vitro, decreasing the release of pro-inflammatory cytokines through nuclear factor kappa B (NF-κB) and MAPK signaling pathways [63,64]; thus, it would be interesting to correlate both macro- and microglial responses to fully understand the neuroprotective effect of DHF.

## 4. Materials and Methods

### 4.1. Animal Handling

Adult female Sprague-Dawley rats (180–220g) were used for this study. Animals were housed in rooms of the animal house of the University of Murcia temperature-controlled with 12h/12h light/dark cycles and were fed with food ad libitum and water. The University of Murcia ethical animal studies committee approved all experiments (Codes: A13170110, A13170111) and followed the Spanish and European Union Directives for animal experiments and the ARVO Statement for the Use of Animals in Ophthalmic and Vision Research.

### 4.2. Intraorbital Optic Nerve Transection

Rats were anaesthetized with i.p. ketamine (60 mg/Kg bw, Ketolar; Pfizer, Alcobendas, Madrid, Spain) and xylazine (10 mg/kg Rompun; Bayer, Kiel, Germany). In the experimental rats, left intraorbital optic nerve transection (IONT) was performed as described [2,65]. Briefly, the left ON was exposed dissecting the orbita contents through a superior temporal approach. The dura sheath was opened longitudinally, and the ON was divided close to its origin without damaging the retinal vessels running on the inferomedial aspect of the sheath. After the surgery, subcutaneous bruprenorfin (0.1 mg/kg; Buprex, Schering-Plough, Madrid, Spain) was administered as analgesic, the eye fundus was inspected to assess retinal blood flow, and to prevent corneal desiccation, an ophthalmic unguent was applied on both eyes (Tobrex^®^; Alcon S.A., Barcelona, Spain). As control, intact animals were used.

### 4.3. Experimental Design

**Figure 5:**
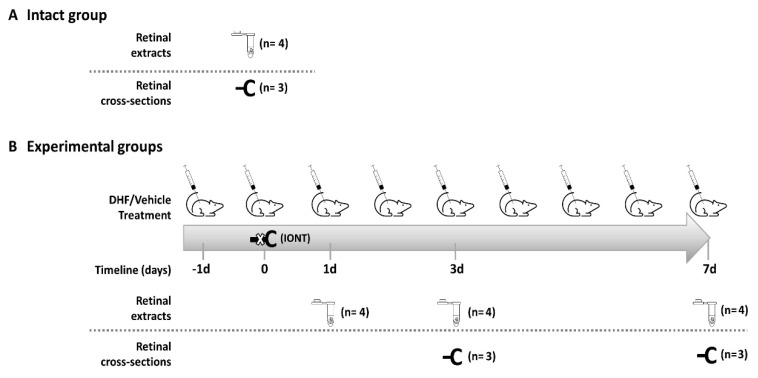
Experimental design: Animals, experimental timeline and analysis of retinas. As control, intact retinas of 2-month-old rats were used (**A**). In the experimental groups (**B**), intraperitoneal injections of DHF (5 mg/kg) or vehicle (0.9 saline in 1% DMSO) were daily administered from one day before intraorbital optic nerve transection (IONT) to the day of processing. In all groups, retinal extracts were used for Western blotting, and retinal cross-sections for immunohistofluorescence. d = days, IONT = intraorbital nerve transection.

The animals were divided in three experimental groups (Figure 5): (i) intact animals, used as control (Figure 5A); (ii) DHF-treated group (dose 5 mg/kg, diluted in vehicle) administered in 1 intraperitoneal (i.p.) injection the day before IONT and 1 i.p. injection/day until processing; and iii) vehicle-treated group (0.9 NaCl containing 1% DMSO) following the same administration regime of the DHF-treated group. These last two groups were processed at 1, 3 and 7 days after IONT (Figure 5B).

Animals were sacrificed with an i.p. overdose of barbituric (Dolethal, Vetoquinol^®^, Especialidades Veterinarias S.A., Madrid, Spain). For Western blotting, retinas were freshly dissected and frozen in dry ice (n = 4/group and time-point). For retinal cross-sections, animals were first perfused and eye cups were prepared for cryostate sectioning of 15 µm thick as previously described (n = 3/group and time-point) [4].

### 4.4. Western Blotting

Retinas were homogenized in 300 µL lysis buffer (PRO-PREPTM Protein Extraction Solution, Intron Biotechnology Inc. Cat. No. 17081), and the amount of protein was measured using a bicinchoninic acid test (B9643 Sigma-Aldrich, Madrid, Spain) with cupric sulfate (CuSO4, 7758-98-7 Sigma-Aldrich). Four individual samples of each group were mixed as a pool sample, and samples were run on 1% SDS-PAGE, transferred to a nitrocellulose membrane and incubated with the following protein specific antibodies overnight at 4 °C: rabbit anti-p-AKT (at serine 473) (1:200, 4060T Cell Signaling, Danvers, MA, USA), rabbit anti-AKT (1:1000, Cell Signalling, 9275S), rabbit anti-p-p42/44 MAPK (ERK1/2, Thr202/Tyr204, 1:100, Cell Signalling, 4370S) and rabbit anti-p42/44 MAPK (Thr202/Tyr204, 1:1000, Cell Signalling, 4695S, which detects endogenous levels of total p44/42 MAP kinase (Erk1/Erk2) protein). Donkey anti-rabbit antibody conjugated to horseradish peroxidase (HRP) (1:5000; Santa Cruz Biotechnologies, Dallas, TX, USA) was used for secondary detection, and samples were visualized by chemiluminescence (Enhanced Chemo Luminiscence [ECL]; Amersham GE Healthcare Europe GmBH). The signal was acquired with an Image LAS 500 (Amersham GE Healthcare Europe GmbH). Westerns were replicated three times, and the protein concentration was calculated considering the intact retinas as 1. As a loading control, β-actin (mouse anti-β Actin HRP conjugated 1:5000, Abcam ab49900) detection was carried out.

### 4.5. Inmunohistofluorescence and Image Acquisition

Retinal cross-sections were immunodetected as described before (Nadal-Nicolas et al., 2009) with the primary antibodies described above (p-AKT, p-MAPK, AKT and MAPK) and mouse anti-Brn3a IgG1 (1:500, MAB1585) and goat anti-vimentin (1:250, Santa Cruz, Sc-7557). The day after, retinas were rinsed in PBS 0.1% Tx and incubated 2h with the secondary antibodies (1:500 in PBS-2% Tx, donkey anti-goat IgG Alexa 594, donkey anti-mouse IgG1 Alexa 594 and donkey anti-rabbit IgG Alexa 488; Molecular Probes Thermo-Fisher, Madrid, Spain), rinsed in PBS mounted with DAPI (Vectashield, Vector laboratories, Palex Medical, Barcelona, Spain).

Images of cross-sectioned retinas were acquired using an epifluorescence microscope (Leica DM4 B, Germany) at 20× magnification controlled by the software LAS X (Leica Application Suite) and the camera Leica (Microsystems Heidelberg GmBH, Germany).To allow qualitative comparisons between the antibodies used in terms of signal intensity, the acquisition settings were the same for all conditions and compared to intact retinas.

### 4.6. Statistics

Statistical comparisons between different groups were done use non-parametric test (ANOVA Kruskal–Wallis post-hoc tests) with the software Graph Pad Prism. A value of *p* < 0.05 was considered statistically significant.

## 5. Conclusions

Using an in vivo adult rat model of retinal injury, we report for the first time the involvement of TrkB-activated signaling pathways in the DHF-afforded protection of RGCs against IONT. Our studies suggest the involvement of the TrkB-activated signaling pathways phosphatidylinositol 3-kinase/protein kinase B (PI3K/AKT) and mitogen-activated protein kinases extracellular signal regulated kinases 1 and 2 (MAPK/ERK; ERK1/2). Moreover, our results indicate an important role for RGCs and Müller cells as effectors in the neuroprotection process.

## Abbreviations

AKT	Protein kinase B
BDNF	Brain-derived neurotrophic factor
CNS	Central nervous system
d	Days post-lesion
DHF	7,8-Dihydroxyflavone
ERK	Extracellular signal-regulated kinase
GCL	Ganglion cell layer
HRP	Horseradish peroxidase
IONT	Intraorbital nerve transection
MAPK	Mitogen-activated protein kinase
MC	Müller cells
NF-kB	Nuclear factor kappa B
NMDA	N-Methyl-D-aspartic acid
OHT	Ocular hypertension
PI3K	Phosphatidylinositol 3 kinase
PLC-Υ	Phospholipase-C
PRL	Photoreceptor layer
RGC	Retinal ganglion cell
RNFL	Retinal nerve fiber layer
ROS	Reactive oxygen species
RPE	Retinal pigment epithelium

## References

[1] Vidal-Sanz M., Nadal-Nicolas F.M., Valiente-Soriano F.J., Agudo-Barriuso M., Villegas-Perez M.P. (2015). Identifying specific RGC types may shed light on their idiosyncratic responses to neuroprotection. Neural. Regen. Res..

[2] Villegas-Perez M.P., Vidal-Sanz M., Rasminsky M., Bray G.M., Aguayo A.J. (1993). Rapid and protracted phases of retinal ganglion cell loss follow axotomy in the optic nerve of adult rats. J. Neurobiol..

[3] Agudo M., Perez-Marin M.C., Sobrado-Calvo P., Lonngren U., Salinas-Navarro M., Canovas I., Nadal-Nicolas F.M., Miralles-Imperial J., Hallbook F., Vidal-Sanz M. (2009). Immediate upregulation of proteins belonging to different branches of the apoptotic cascade in the retina after optic nerve transection and optic nerve crush. Investig. Ophthalmol. Vis. Sci..

[4] Nadal-Nicolas F.M., Jimenez-Lopez M., Sobrado-Calvo P., Nieto-Lopez L., Canovas-Martinez I., Salinas-Navarro M., Vidal-Sanz M., Agudo M. (2009). Brn3a as a marker of retinal ganglion cells: Qualitative and quantitative time course studies in naive and optic nerve-injured retinas. Investig. Ophthalmol. Vis. Sci..

[5] Vidal-Sanz M., Galindo-Romero C., Valiente-Soriano F.J., Nadal-Nicolas F.M., Ortin-Martinez A., Rovere G., Salinas-Navarro M., Lucas-Ruiz F., Sanchez-Migallon M.C., Sobrado-Calvo P. (2017). Shared and differential retinal responses against optic nerve injury and ocular hypertension. Front. Neurosci..

[6] Galindo-Romero C., Aviles-Trigueros M., Jimenez-Lopez M., Valiente-Soriano F.J., Salinas-Navarro M., Nadal-Nicolas F., Villegas-Perez M.P., Vidal-Sanz M., Agudo-Barriuso M. (2011). Axotomy-induced retinal ganglion cell death in adult mice: Quantitative and topographic time course analyses. Exp. Eye Res..

[7] Galindo-Romero C., Valiente-Soriano F.J., Jimenez-Lopez M., Garcia-Ayuso D., Villegas-Perez M.P., Vidal-Sanz M., Agudo-Barriuso M. (2013). Effect of brain-derived neurotrophic factor on mouse axotomized retinal ganglion cells and phagocytic microglia. Investig. Ophthalmol. Vis. Sci..

[8] Lucas-Ruiz F., Galindo-Romero C., Rodriguez-Ramirez K.T., Vidal-Sanz M., Agudo-Barriuso M. (2019). Neuronal death in the contralateral un-injured retina after unilateral axotomy: Role of microglial cells. Int. J. Mol. Sci..

[9] Sanchez-Migallon M.C., Nadal-Nicolas F.M., Jimenez-Lopez M., Sobrado-Calvo P., Vidal-Sanz M., Agudo-Barriuso M. (2011). Brain derived neurotrophic factor maintains Brn3a expression in axotomized rat retinal ganglion cells. Exp. Eye Res..

[10] Sanchez-Migallon M.C., Valiente-Soriano F.J., Salinas-Navarro M., Nadal-Nicolas F.M., Jimenez-Lopez M., Vidal-Sanz M., Agudo-Barriuso M. (2018). Nerve fibre layer degeneration and retinal ganglion cell loss long term after optic nerve crush or transection in adult mice. Exp. Eye Res..

[11] Sanchez-Migallon M.C., Valiente-Soriano F.J., Nadal-Nicolas F.M., Vidal-Sanz M., Agudo-Barriuso M. (2016). Apoptotic retinal ganglion cell death after optic nerve transection or crush in mice: Delayed RGC Loss with BDNF or a caspase 3 inhibitor. Investig. Ophthalmol. Vis. Sci..

[12] Lonngren U., Napankangas U., Lafuente M., Mayor S., Lindqvist N., Vidal-Sanz M., Hallbook F. (2006). The growth factor response in ischemic rat retina and superior colliculus after brimonidine pre-treatment. Brain Res. Bull..

[13] Lindqvist N., Peinado-Ramonn P., Vidal-Sanz M., Hallbook F. (2004). GDNF, Ret, GFRalpha1 and 2 in the adult rat retino-tectal system after optic nerve transection. Exp. Neurol..

[14] Valiente-Soriano F.J., Nadal-Nicolas F.M., Salinas-Navarro M., Jimenez-Lopez M., Bernal-Garro J.M., Villegas-Perez M.P., Agudo-Barriuso M., Vidal-Sanz M. (2015). BDNF Rescues RGCs but not intrinsically photosensitive RGCs in ocular hypertensive albino rat retinas. Investig. Ophthalmol. Vis. Sci..

[15] Vidal-Sanz M., Lafuente M., Sobrado-Calvo P., Selles-Navarro I., Rodriguez E., Mayor-Torroglosa S., Villegas-Perez M.P. (2000). Death and neuroprotection of retinal ganglion cells after different types of injury. Neurotox. Res..

[16] Vidal-Sanz M., Lafuente M.P., Mayor S., de Imperial J.M., Villegas-Perez M.P. (2001). Retinal ganglion cell death induced by retinal ischemia: Neuroprotective effects of two alpha-2 agonists. Surv. Ophthalmol..

[17] Lafuente M.P., Villegas-Perez M.P., Sobrado-Calvo P., Garcia-Aviles A., Miralles de Imperial J., Vidal-Sanz M. (2001). Neuroprotective effects of alpha(2)-selective adrenergic agonists against ischemia-induced retinal ganglion cell death. Investig. Ophthalmol. Vis. Sci..

[18] Boia R., Ruzafa N., Aires I.D., Pereiro X., Ambrosio A.F., Vecino E., Santiago A.R. (2020). Neuroprotective strategies for retinal ganglion cell degeneration: Current status and challenges ahead. Int. J. Mol. Sci..

[19] Mansour-Robaey S., Clarke D.B., Wang Y.C., Bray G.M., Aguayo A.J. (1994). Effects of ocular injury and administration of brain-derived neurotrophic factor on survival and regrowth of axotomized retinal ganglion cells. Proc. Natl. Acad. Sci. USA.

[20] Cheng L., Sapieha P., Kittlerova P., Hauswirth W.W., Di Polo A. (2002). TrkB gene transfer protects retinal ganglion cells from axotomy-induced death in vivo. J. Neurosci..

[21] Di Polo A., Aigner L.J., Dunn R.J., Bray G.M., Aguayo A.J. (1998). Prolonged delivery of brain-derived neurotrophic factor by adenovirus-infected Muller cells temporarily rescues injured retinal ganglion cells. Proc. Natl. Acad. Sci. USA.

[22] Peinado-Ramon P., Salvador M., Villegas-Perez M.P., Vidal-Sanz M. (1996). Effects of axotomy and intraocular administration of NT-4, NT-3, and brain-derived neurotrophic factor on the survival of adult rat retinal ganglion cells. A quantitative in vivo study. Investig. Ophthalmol. Vis. Sci..

[23] Valiente-Soriano F.J., Ortin-Martinez A., Di Pierdomenico J., Garcia-Ayuso D., Gallego-Ortega A., Miralles de Imperial-Ollero J.A., Jimenez-Lopez M., Villegas-Perez M.P., Wheeler L.A., Vidal-Sanz M. (2019). Topical brimonidine or intravitreal BDNF, CNTF, or bFGF protect cones against phototoxicity. Transl. Vis. Sci. Technol..

[24] Parrilla-Reverter G., Agudo M., Sobrado-Calvo P., Salinas-Navarro M., Villegas-Perez M.P., Vidal-Sanz M. (2009). Effects of different neurotrophic factors on the survival of retinal ganglion cells after a complete intraorbital nerve crush injury: A quantitative in vivo study. Exp. Eye Res..

[25] Rovere G., Nadal-Nicolas F.M., Sobrado-Calvo P., Garcia-Bernal D., Villegas-Perez M.P., Vidal-Sanz M., Agudo-Barriuso M. (2016). Topical treatment with bromfenac reduces retinal gliosis and inflammation after optic nerve crush. Investig. Ophthalmol. Vis. Sci..

[26] Ortin-Martinez A., Valiente-Soriano F.J., Garcia-Ayuso D., Alarcon-Martinez L., Jimenez-Lopez M., Bernal-Garro J.M., Nieto-Lopez L., Nadal-Nicolas F.M., Villegas-Perez M.P., Wheeler L.A. (2014). A novel in vivo model of focal light emitting diode-induced cone-photoreceptor phototoxicity: Neuroprotection afforded by brimonidine, BDNF, PEDF or bFGF. PLoS ONE.

[27] Kaplan D.R., Miller F.D. (2000). Neurotrophin signal transduction in the nervous system. Curr. Opin. Neurobiol..

[28] Yamada K., Nabeshima T. (2003). Brain-derived neurotrophic factor/TrkB signaling in memory processes. J. Pharmacol. Sci..

[29] Nakazawa T., Tamai M., Mori N. (2002). Brain-derived neurotrophic factor prevents axotomized retinal ganglion cell death through MAPK and PI3K signaling pathways. Investig. Ophthalmol. Vis. Sci..

[30] Park H., Poo M.M. (2013). Neurotrophin regulation of neural circuit development and function. Nat. Rev. Neurosci..

[31] Baydyuk M., Xu B. (2014). BDNF signaling and survival of striatal neurons. Front. Cell Neurosci..

[32] Gonzalez A., Moya-Alvarado G., Gonzalez-Billaut C., Bronfman F.C. (2016). Cellular and molecular mechanisms regulating neuronal growth by brain-derived neurotrophic factor. Cytoskeleton.

[33] Yoshii A., Constantine-Paton M. (2010). Postsynaptic BDNF-TrkB signaling in synapse maturation, plasticity, and disease. Dev. Neurobiol..

[34] Cappoli N., Tabolacci E., Aceto P., Dello Russo C. (2020). The emerging role of the BDNF-TrkB signaling pathway in the modulation of pain perception. J. Neuroimmunol..

[35] Chitranshi N., Gupta V., Kumar S., Graham S.L. (2015). Exploring the molecular interactions of 7,8-dihydroxyflavone and its derivatives with TrkB and VEGFR2 proteins. Int. J. Mol. Sci..

[36] Liu X., Obianyo O., Chan C.B., Huang J., Xue S., Yang J.J., Zeng F., Goodman M., Ye K. (2014). Biochemical and biophysical investigation of the brain-derived neurotrophic factor mimetic 7,8-dihydroxyflavone in the binding and activation of the TrkB receptor. J. Biol. Chem..

[37] Cerquone Perpetuini A., Mathoux J., Kennedy B.N. (2019). The potential of small molecule brain-derived neurotrophic factor: Mimetics to treat inherited retinal degeneration. Neural Regen. Res..

[38] Liu X., Qi Q., Xiao G., Li J., Luo H.R., Ye K. (2013). O-methylated metabolite of 7,8-dihydroxyflavone activates TrkB receptor and displays antidepressant activity. Pharmacology.

[39] Zhang Z., Liu X., Schroeder J.P., Chan C.B., Song M., Yu S.P., Weinshenker D., Ye K. (2014). 7,8-dihydroxyflavone prevents synaptic loss and memory deficits in a mouse model of Alzheimer’s disease. Neuropsychopharmacology.

[40] Emili M., Guidi S., Uguagliati B., Giacomini A., Bartesaghi R., Stagni F. (2020). Treatment with the flavonoid 7,8-dihydroxyflavone: A promising strategy for a constellation of body and brain disorders. Crit. Rev. Food Sci. Nutr..

[41] Gupta V.K., You Y., Li J.C., Klistorner A., Graham S.L. (2013). Protective effects of 7,8-dihydroxyflavone on retinal ganglion and RGC-5 cells against excitotoxic and oxidative stress. J. Mol. Neurosci..

[42] Huang H.M., Huang C.C., Tsai M.H., Poon Y.C., Chang Y.C. (2018). Systemic 7,8-dihydroxyflavone treatment protects immature retinas against hypoxic-ischemic injury via Muller glia regeneration and MAPK/ERK activation. Investig. Ophthalmol. Vis. Sci..

[43] Fang Y.Y., Luo M., Yue S., Han Y., Zhang H.J., Zhou Y.H., Liu K., Liu H.G. (2021). 7,8-Dihydroxyflavone protects retinal ganglion cells against chronic intermittent hypoxia-induced oxidative stress damage via activation of the BDNF/TrkB signaling pathway. Sleep Breath.

[44] Liu C., Chan C.B., Ye K. (2016). 7,8-dihydroxyflavone, a small molecular TrkB agonist, is useful for treating various BDNF-implicated human disorders. Transl. Neurodegener..

[45] Vidal-Villegas B., Di Pierdomenico J., Gallego-Ortega A., Galindo-Romero C., Martinez-de-la-Casa J.M., Garcia-Feijoo J., Villegas-Perez M.P., Vidal-Sanz M. (2021). Systemic treatment with 7,8-Dihydroxiflavone activates TtkB and affords protection of two different retinal ganglion cell populations against axotomy in adult rats. Exp. Eye Res..

[46] Jang S.W., Liu X., Yepes M., Shepherd K.R., Miller G.W., Liu Y., Wilson W.D., Xiao G., Blanchi B., Sun Y.E. (2010). A selective TrkB agonist with potent neurotrophic activities by 7,8-dihydroxyflavone. Proc. Natl. Acad. Sci. USA.

[47] Chitranshi N., Dheer Y., Abbasi M., You Y., Graham S.L., Gupta V. (2018). Glaucoma pathogenesis and neurotrophins: Focus on the molecular and genetic basis for therapeutic prospects. Curr. Neuropharmacol..

[48] Almasieh M., Wilson A.M., Morquette B., Cueva Vargas J.L., Di Polo A. (2012). The molecular basis of retinal ganglion cell death in glaucoma. Prog. Retin. Eye Res..

[49] Liu X., Chan C.B., Jang S.W., Pradoldej S., Huang J., He K., Phun L.H., France S., Xiao G., Jia Y. (2010). A synthetic 7,8-dihydroxyflavone derivative promotes neurogenesis and exhibits potent antidepressant effect. J. Med. Chem..

[50] Segal R.A., Greenberg M.E. (1996). Intracellular signaling pathways activated by neurotrophic factors. Annu. Rev. Neurosci..

[51] Galan A., Dergham P., Escoll P., de-la-Hera A., D’Onofrio P.M., Magharious M.M., Koeberle P.D., Frade J.M., Saragovi H.U. (2014). Neuronal injury external to the retina rapidly activates retinal glia, followed by elevation of markers for cell cycle re-entry and death in retinal ganglion cells. PLoS ONE.

[52] González-Riquelme M.J., Galindo-Romero C., Lucas-Ruiz F., Martínez-Carmona M., Rodríguez-Ramírez K.T., Cabrera-Maqueda J.M., Norte-Muñoz M., Vidal-Sanz M., Agudo-Barriuso M. (2021). Axonal injuries cast long shadows: Long term glial activation in injured and contralateral retinas after unilateral axotomy. Int. J. Mol. Sci..

[53] Harun-Or-Rashid M., Diaz-DelCastillo M., Galindo-Romero C., Hallbook F. (2015). Alpha2-adrenergic-agonist brimonidine stimulates negative feedback and attenuates injury-induced phospho-ERK and dedifferentiation of chicken muller cells. Investig. Ophthalmol. Vis. Sci..

[54] Harun-Or-Rashid M., Konjusha D., Galindo-Romero C., Hallbook F. (2016). Endothelin B Receptors on primary chicken Muller cells and the human MIO-M1 Muller cell line activate ERK signaling via transactivation of epidermal growth factor receptors. PLoS ONE.

[55] Bringmann A., Pannicke T., Grosche J., Francke M., Wiedemann P., Skatchkov S.N., Osborne N.N., Reichenbach A. (2006). Muller cells in the healthy and diseased retina. Prog. Retin. Eye Res..

[56] Valiente-Soriano F.J., Salinas-Navarro M., Di Pierdomenico J., Garcia-Ayuso D., Lucas-Ruiz F., Pinilla I., Cuenca N., Vidal-Sanz M., Villegas-Perez M.P., Agudo-Barriuso M. (2020). Tracing the retina to analyze the integrity and phagocytic capacity of the retinal pigment epithelium. Sci. Rep..

[57] Sparrow J.R., Hicks D., Hamel C.P. (2010). The retinal pigment epithelium in health and disease. Curr. Mol. Med..

[58] Benn S.C., Woolf C.J. (2004). Adult neuron survival strategies-slamming on the brakes. Nat. Rev. Neurosci..

[59] Mao D., Sun X. (2015). Reactivation of the PI3K/Akt signaling pathway by the bisperoxovanadium compound bpV(pic) attenuates photoreceptor apoptosis in experimental retinal detachment. Investig. Ophthalmol. Vis. Sci..

[60] Chen J., Chua K.W., Chua C.C., Yu H., Pei A., Chua B.H., Hamdy R.C., Xu X., Liu C.F. (2011). Antioxidant activity of 7,8-Dihydroxyflavone provides neuroprotection against glutamate-induced toxicity. Neurosci. Lett..

[61] Cho S.J., Kang K.A., Piao M.J., Ryu Y.S., Fernando P., Zhen A.X., Hyun Y.J., Ahn M.J., Kang H.K., Hyun J.W. (2019). 7,8-Dihydroxyflavone protects high glucose-damaged neuronal cells against oxidative stress. Biomol. Ther..

[62] Nadal-Nicolas F.M., Jimenez-Lopez M., Salinas-Navarro M., Sobrado-Calvo P., Vidal-Sanz M., Agudo-Barriuso M. (2017). Microglial dynamics after axotomy-induced retinal ganglion cell death. J. Neuroinflamm..

[63] Park H.Y., Kim G.Y., Hyun J.W., Hwang H.J., Kim N.D., Kim B.W., Choi Y.H. (2012). 7,8-Dihydroxyflavone exhibits anti-inflammatory properties by downregulating the NF-kappaB and MAPK signaling pathways in lipopolysaccharide-treated RAW264.7 cells. Int. J. Mol. Med..

[64] Park H.Y., Park C., Hwang H.J., Kim B.W., Kim G.Y., Kim C.M., Kim N.D., Choi Y.H. (2014). 7,8-Dihydroxyflavone attenuates the release of pro-inflammatory mediators and cytokines in lipopolysaccharide-stimulated BV2 microglial cells through the suppression of the NF-kappaB and MAPK signaling pathways. Int. J. Mol. Med..

[65] Vidal-Sanz M., Bray G.M., Villegas-Perez M.P., Thanos S., Aguayo A.J. (1987). Axonal regeneration and synapse formation in the superior colliculus by retinal ganglion cells in the adult rat. J. Neurosci..

